# Supplementary material for: Super-geometric electron focusing on the hexagonal Fermi surface of PdCoO2
Source: Nat Commun. 2019 Nov 8;10:5081. doi: 10.1038/s41467-019-13020-9 (PMC6841680; doi:10.1038/s41467-019-13020-9)
Supplement: Supplementary file 1 — Supplementary Information [file 41467_2019_13020_MOESM1_ESM.pdf]

Supplementary Information to "Bachmann *et al.*, Super-geometric electron focusing on the hexagonal Fermi surface of $PdCoO_2$"

Supplementary Note 1. Synthesis and characterization of $PdCoO_2$ crystals

Single crystals were grown in an evacuated quartz ampule with a mixture of $PdCl_2$ and CoO by the following methathetical reaction[35]: $PdCl_2 + 2CoO \rightarrow 2PdCoO_2 + CoCl_2$. The ampule was heated at 1000 °C for 12 hours and stayed at 700-750 °C for 5 days. In order to remove $CoCl_2$, the resultant product was washed with distilled water and ethanol.

The orientation of the crystallographic axes was determined using the back-reflection Laue method. It was consistently found in over 5 crystals, that the in-plane *a*- and *b*-axes are rotated 90 degrees with respect to the hexagonal growth edges (see Fig. 2a). The out-of-plane c-axis lies perpendicular to the crystal platelets.

Quantitative energy dispersive X-ray spectroscopy (EDS), displayed in figure S1a, was used to confirm the elemental composition of the delafossite crystals using the AZtec software platform from Oxford Instruments. Typically, the oxygen concentration is severely underestimated due to a wrong carbon coating thickness, since carbon has an absorption edge near oxygen and heavily absorbs oxygen x-rays. Therefore, if the oxygen is fixed by stoichiometry to 2 ions, Pd and Co are found in equal atomic concentration.

Additionally, we present the Shubnikov-de Haas oscillations measured in a FIB-defined $PdCoO_2$ transport bar, with current flowing along the crystallographic *a*-axis in an out-of-plane magnetic field ($B \parallel c$) at 2K. The clear observation of quantum oscillations demonstrates that the high crystalline quality of the crystals after FIB sample fabrication. The observed frequencies F_1 = 28.9 kT and F_2 = 30.2kT, and masses $m_1 = 1.65 m_e$ and $m_2 = 1.73 m_e \pm 0.02$ agree well with the reported values[14] for bulk samples.

The residual resistance ratios extracted from the data in Fig. 2d are 457, 459 and 355 for V_B, V_C and V_D respectively. Although the size of the overall device exceeds the mean free path, ballistic effects at low temperatures may lead to a correction of the measured non-local voltage.

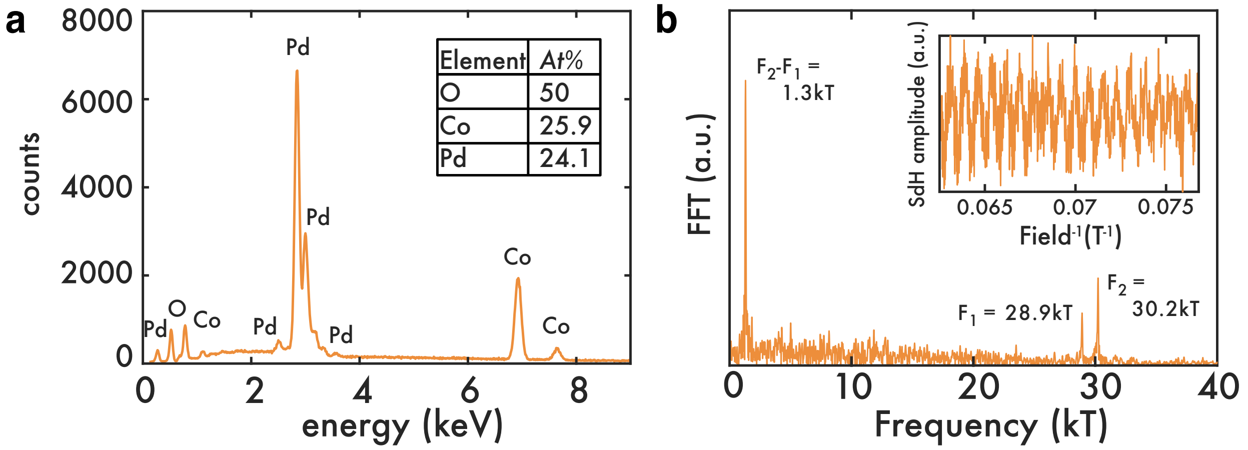

Supplementary Figure 1: Characterization of FIB microstructured $PdCoO_2$ crystals.

a) SEM-EDS spectrum of a $PdCoO_2$ crystal. Insert: Elemental analysis report after fixing the oxygen content by stoichiometry to 2.

b) Fast Fourier transform (FFT) of the Shubnikov-de Haas (SdH) oscillations after a smooth back ground subtraction (inset). The main frequencies F_1, F_2, as well as their difference frequency F_2-F_1 agree well with reported values in literature[14].

Supplementary Note 2. FIB fabrication of point-like injections nozzles

During this investigation, a total of 7 $PdCoO_2$ crystal platelets were structured into TEF devices. These included 5 nozzle sets oriented along the 2-beam direction, 2 nozzle sets oriented along the 3-beam direction and 3 nozzle sets oriented 11 degrees away from the 2-beam and 3-beam direction respectively. The measurement results of these devices reproduce the data shown in figure 3. The following image series displays the device fabrication process used for a single crystal into which one set of nozzles oriented along the 2-beam direction was cut. The fabrication approach described here can be used to fabricate nozzle along any desired in-plane direction of the crystal.

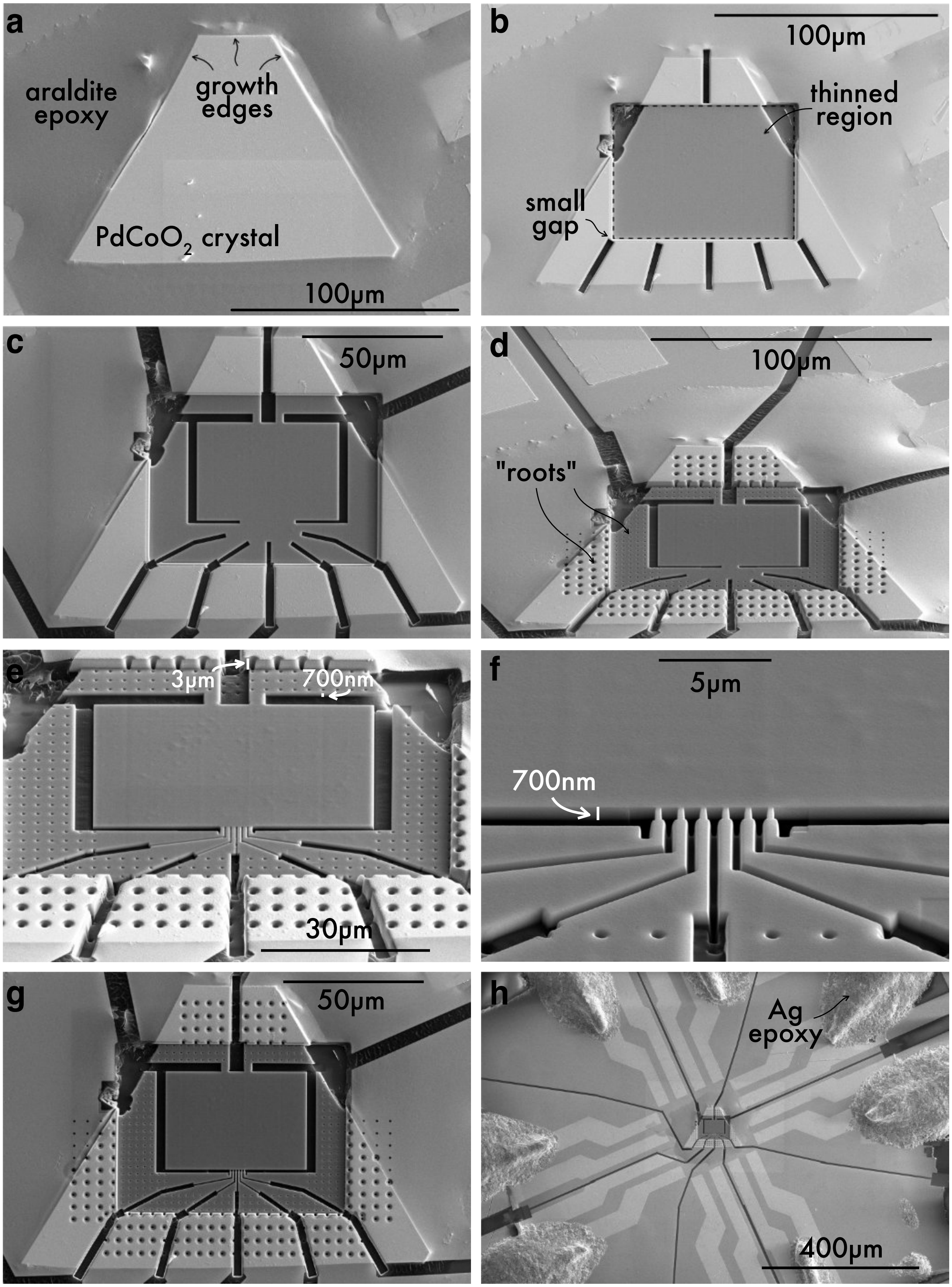

Supplementary Figure 2: Step-by-step overview of the fabrication of a ballistic delafossite device using a Ga-based FIB.

a) A $PdCoO_2$ crystal is fixed onto a sapphire substrate (1.6 x 1.6 x 0.4 mm^3) with 5 min araldite® rapid epoxy, which is cured for 1 hour at 100°C on a hot plate. Care is taken to select a crystal with as little step edges as possible and with well-defined hexagonal growth edges, such that the crystallographic orientation can be directly determined.

b) The crystal is thinned down in the center to a final thickness of less than 1 μm (here 700 nm), using Ga^{2+} ions at 30kV, cutting a rectangle pattern with a current of 65nA, 1 μs dwell time and the "dynamic all directions" scan option. Thinning down the crystal is a necessary step to fabricate narrow, closely spaced nozzles later on. Further ~10μm wide rectangular cuts are made using 65 nA through the remaining thick parts of the crystal to define current and voltage contacts. A small gap is left to reduce re-deposition in the central area.

c) Rectangular cuts are patterned with 2.5 – 9.3 nA in the central region, which define a rectangular measurement region. The sides of the rectangle are polished with 2.5 nA under an angle of +1 degree with respect to the normal milling direction to obtain flat boundaries.

d) In order to ensure a homogenous current flow between all palladium layers despite having a top current injection, holes are patterned through the entire depth of the crystal with a current of 47nA and 2ms dwell time. At the inner edge of these 'root'-like features, the amorphous FIB-damage layer and re-deposition couples the individual layers together and increases the interlayer conductance. Roots are also milled into the central part of the device using a current of 2.5nA and 2ms dwell time.

e) The constrictions leading up to the nozzles are patterned with 80pA. Making long and thin constrictions is favorable, as they act as long flexures and reduce mechanical cracking of then nozzles due to strain from differential thermal contraction while cooling down.

f) The nozzles are cut using an array of cleaning cross section (CCS) cuts at 40pA, cut under an angle of 1degree. Initially the nozzles to a width of about 500nm and are then sequentially thinned down with CCSs until the final width of the nozzle is achieved.

g) Overview of the final device. If the nozzles are thinner than 350 nm, a second layer of 5 min araldite epoxy is added on top of the finished device and cured at room temperature for 24 hours. This reduces the substrate strain during cooldown and avoids nozzle fracture.

h) Final device on sapphire substrate. Silver wires were attached using Epotek EE129-4 silver epoxy and cured at 100°C for 1 hour. A 100 nm thick layer of sputtered Au connects the pre-evaporated gold leads on the substrate with the crystal device.

Supplementary Note 3. Current dependence

As a result of the extremely high in-plane conductivity of $PdCoO_2$ at low temperatures, comparatively large measurement currents are necessary in order to record clean signatures of the TEF effect. The typical measurement current used for the devices presented here is on the order of 1mA. In order to exclude the occurrence of self-heating effects, we have performed the same TEF measurement for a range of current spanning nearly two orders of magnitude (25μA, 100μA, 1mA, 2mA), thus varying the input power P nearly four orders of magnitude. As presented in figure S3a, the resulting position, height and width of the TEF peaks do not undergo any measurable change. We therefor conclude that the system is in the linear response regime.

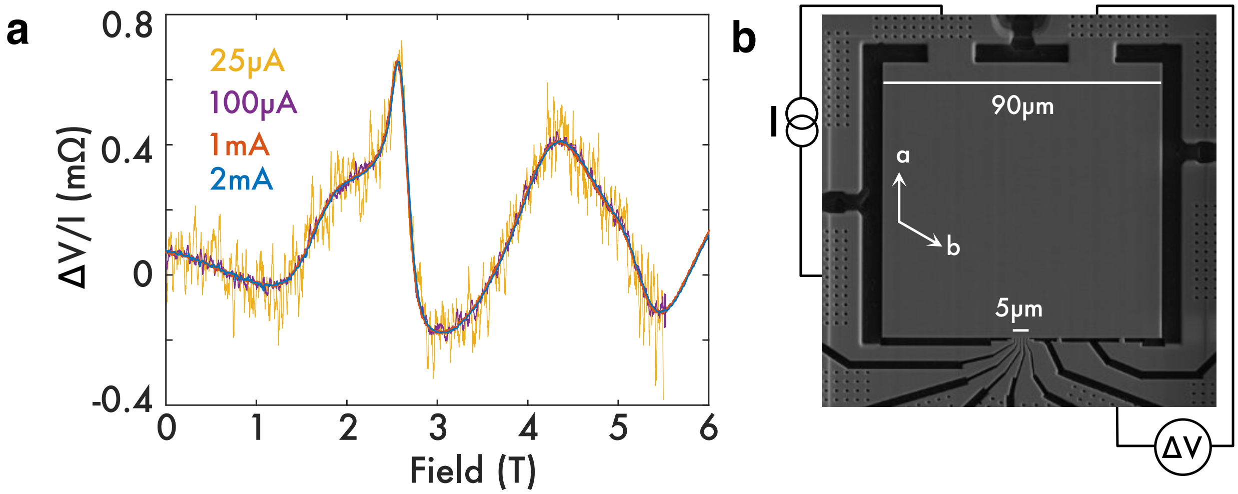

Supplementary Figure 3: Current dependence of the TEF signal.

a) Four currents (25μA, 100μA, 1mA, 2mA) spanning nearly two orders of magnitude were applied to the device shown in panel b. No significant change between the individual TEF signal is observable.

b) A current was sources from the left most nozzle to the top left electrode, while the voltage was measured between a nozzle separated by 5μm from the injection nozzle and the top right electrode. The widths of the nozzles is around 280nm.

Supplementary Note 4. Reproducibility

In the following we compare the measured TEF signal for 3 distinct devices fabricated from 3 different crystals. In order to compare the measured signals, we divide the voltage by the applied current as well as the device thickness, summarized in the table below. The observed differences in peak height and position (see fig. S4a) can partially be accounted for taking the varying nozzle widths into consideration. Additionally, the shape of the nozzle influences the degree of collimation the ejected electron beam and can therefore change the height of the focusing peak. We conclude that the key signatures of the TEF signals are highly reproducible, while details may depend on the specific device geometry implemented.

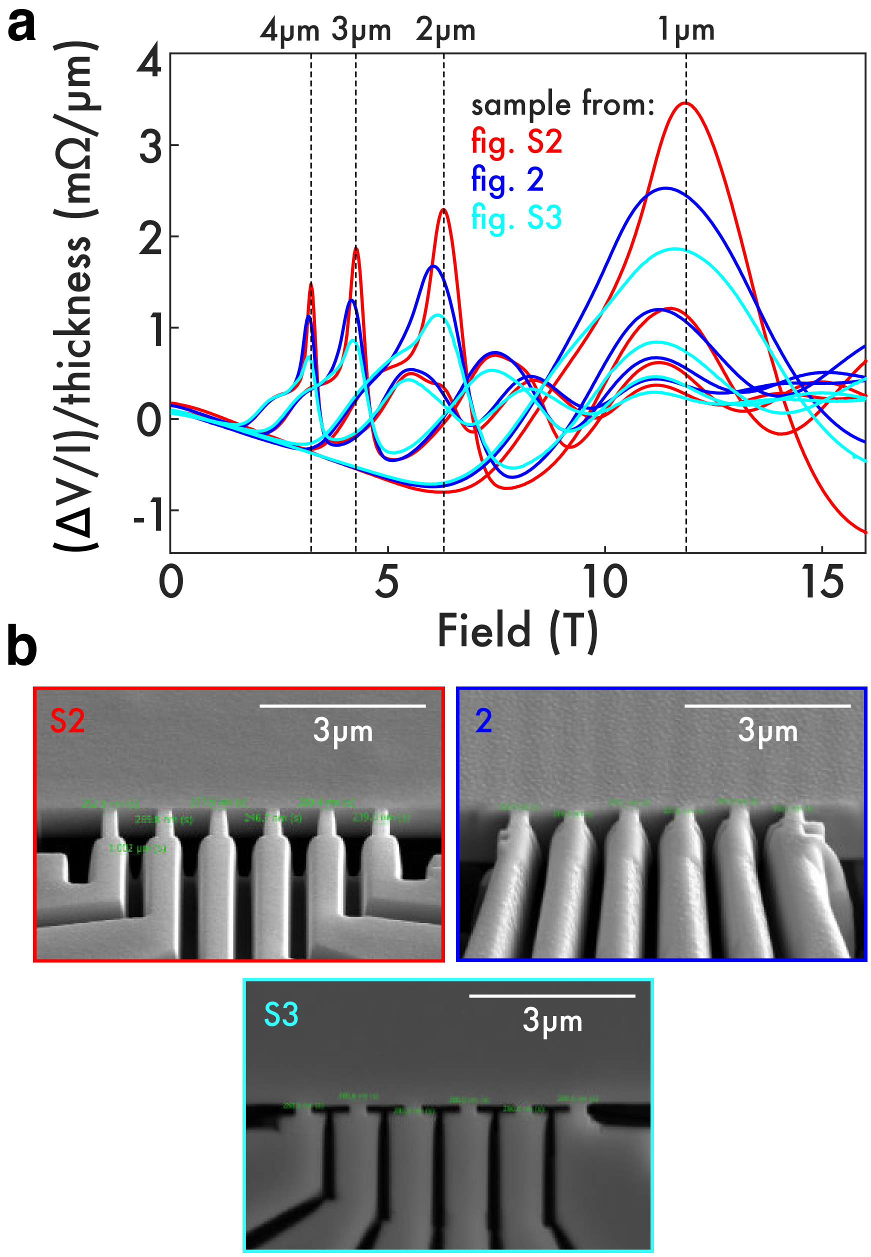

Supplementary Figure 4: Comparison of the TEF signal measured in 3 different devices.

a) The devices corresponding to the data presented here are displayed in figure 2 (blue), S2 (red) and S3 (cyan) respectively. The peaks of the TEF signals, indicated by dashed lines, are located at 3.22T, 4.25T, 6.28T and 11.86T, for nozzle separations of 4, 3, 2, and 1μm.

b) Close up comparison of the various nozzle shapes and widths for the three devices. The various nozzle widths and thicknesses are summarized in the following table.

Device in presented in figure	thickness	nozzle width
2	1.2μm	290 - 340 nm
S2	0.69μm	200 - 260nm
S3	1.23μm	280 – 290 nm

Supplementary Table 1: Overview of the device dimensions of the samples presented in figure S4.

Supplementary Note 5. Long range Focusing

Here we present the TEF signal of the furthest spaced nozzle pair which we have fabricated. The distance between these nozzles (1 and 8 from figure 2) is 35μm, which amounts to a path length of approximately 53μm through the sample (sketched in blue in panel a). As a comparison, we also show the TEF signal of a nozzle pair which is separated by a distance of 15μm. The measurements performed at 2K are presented in panel b, in which up to 8 TEF peaks are clearly identifiably. This demonstrates the length scale over which ballistic effects are still observable.

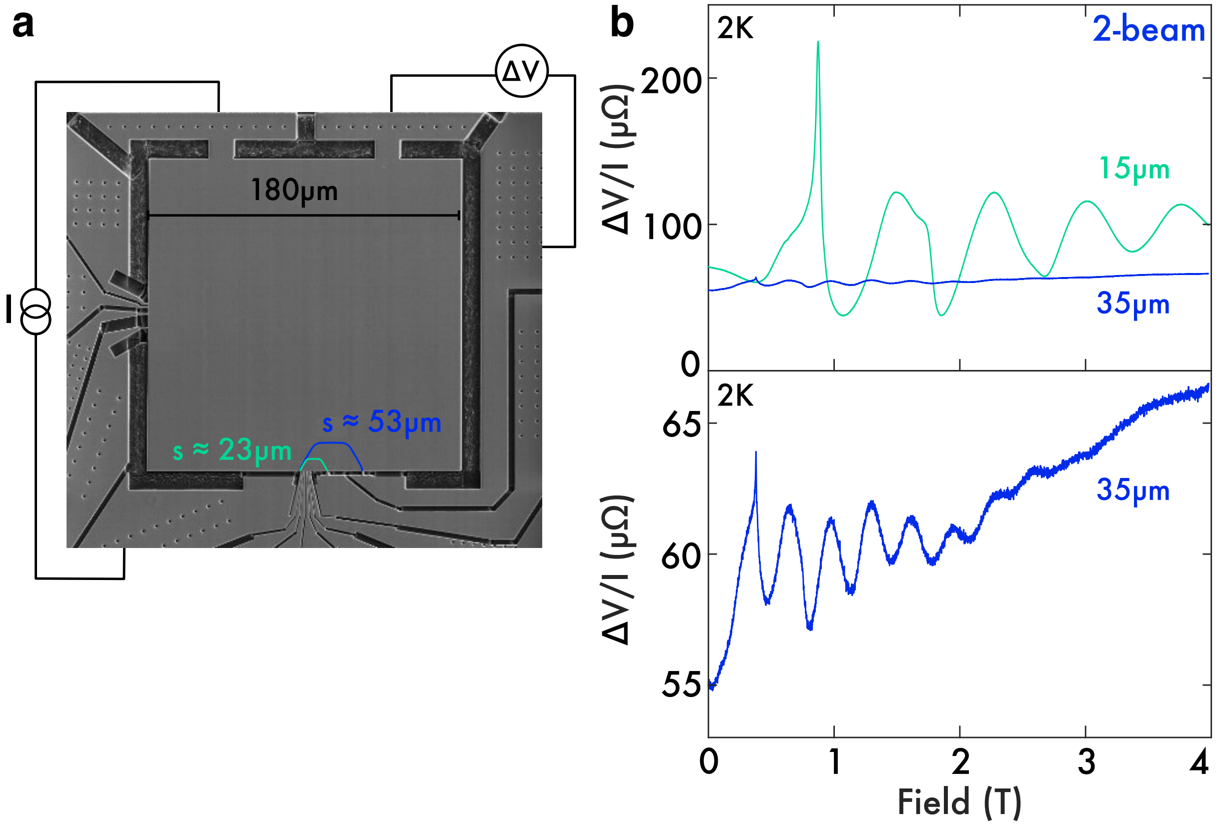

Supplementary Figure 5: TEF across distances greater than the mean-free-path.

a) Measurement setup and propagation path through the sample. Along the 2-beam direction a nozzle separation of L_1=15 μm and L_2=35 μm corresponds to a path length ($s = \frac{3}{2}L$ in a perfect hexagon) through the device of $s_1 \approx$ 23 μm and $s_2 \approx$ 53 μm respectively.

b) Measured voltage ΔV divided by the applied current I = 6 mA as a function of transverse magnetic field at a temperature of 1.8 K. Top: Direct comparison of the signals of a 15μm and 35 μm separated nozzle pair. Bottom: Magnified signal of the 35 μm spaced nozzle pair. The double peak feature as well as 7 higher harmonic peaks are detectable.

Supplementary Note 6. Peak shape analysis from ballistic simulations

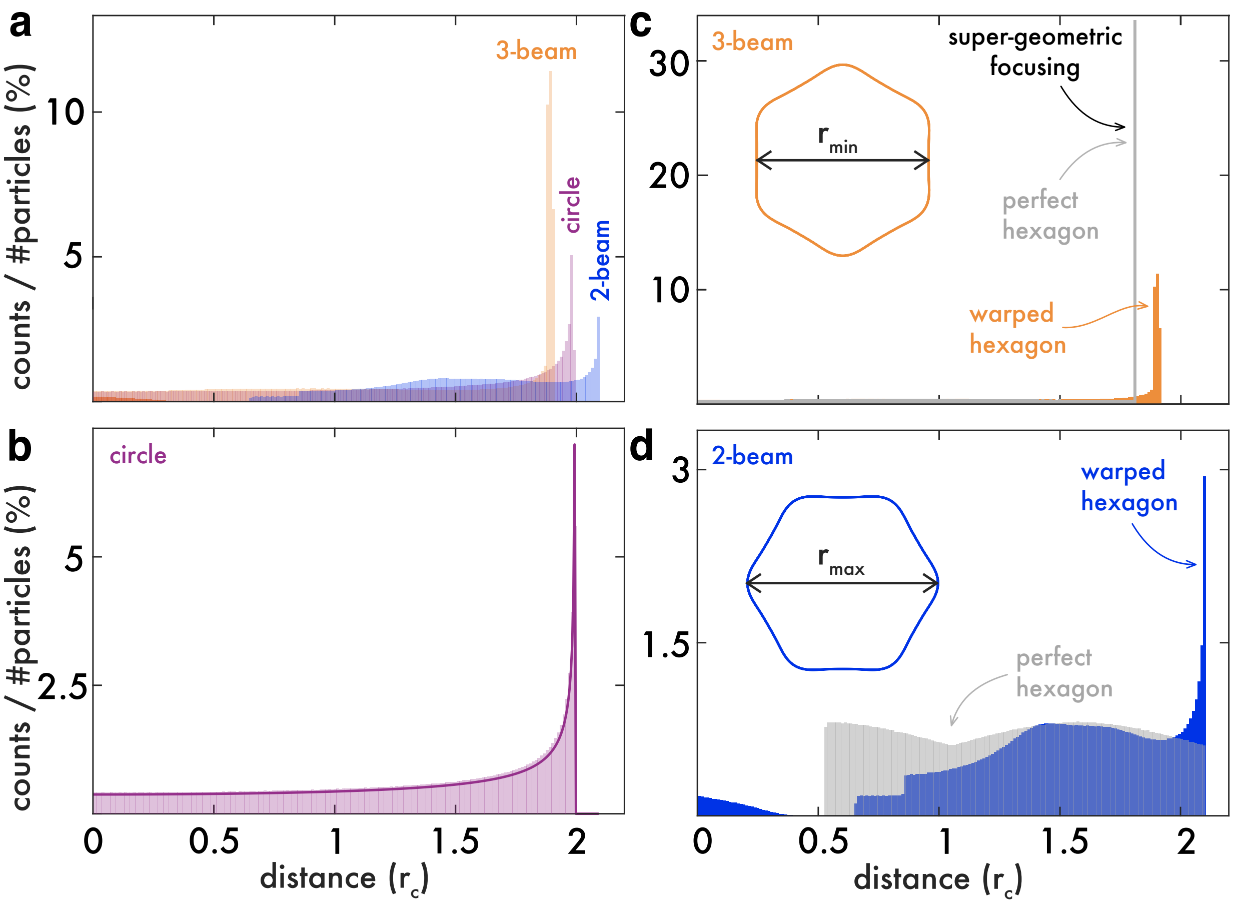

Supplementary Figure 6: Comparison of the peak shapes for a circular, perfect and a realistic model of the rounded hexagonal Fermi surface

a) Comparison of the focusing spectra and peak heights of TEF in a circular FS and measured along the 3-beam and 2-beam direction in $PdCoO_2$. The cyclotron diameter is a factor $\frac{\sqrt{3}}{2}$ smaller in the 3-beam direction compared to the 2-beam direction, corresponding to the difference between inradius and circumradius of a hexagon. The diameter of the circular FS is expressed relative to the circumradius of the hexagon and was chosen smaller for clarity. Compared to a circular FS the 3-beam direction has an enhanced peak, while the main peak along the 2-beam direction is reduced and has a second broad hump.

b) In a circular FS the simulated focusing spectrum (shaded purple) diverges. For the mathematical derivation c.f. methods S10.

c) Comparison of the TEF spectra of a perfect hexagon and a hexagon with warped sides and rounded corners inferred from the FS of $PdCoO_2$ along the 3-beam direction. The perfectly flat edges lead to a geometrical enhancement of height of the focusing peak (so called super-geometric focusing or 'sfocusing'). In a warped hexagon, sfocusing still leads to an increased TEF peak.

d) Along the 2-beam direction, a perfect hexagonal FS does not exhibit any TEF at all, because there are no FS regions that are parallel to the nozzle injection direction. For a hexagon with rounded corners a focusing peak is recovered, analogous to the case of a circular FS.

Supplementary Note 7. Amplitude ratio analysis

The amplitude ratio of subsequent TEF peaks, $q = A_n/A_{n+1}$, is often believed to be a direct probe of the specularity of the boundaries. In this simple picture, the decay of the peak height is equated to the boundary specularity coefficient p. This, however, neglects corrections due to Fermi surface shape. To identify the relation between q and p, we have therefore performed ballistic simulations for a hexagonal FS (3-beam and 2-beam direction) as well as a circular FS.

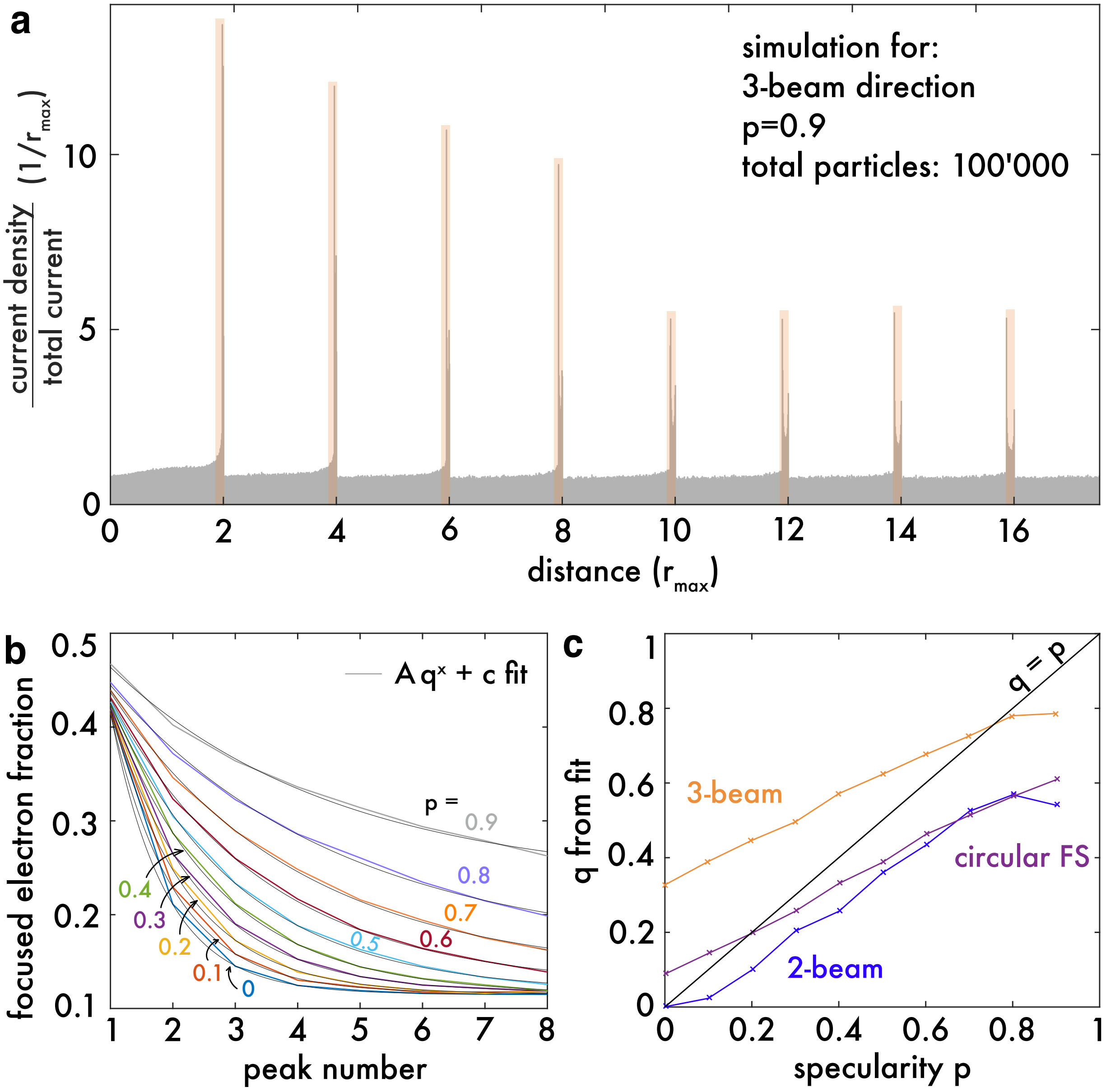

Supplementary Figure 7: Simulations and analysis of TEF peaks for various boundary specularity coefficients.

a) Simulated TEF spectrum along the 3-beam direction for a total of 100'000 particles with an isotropic incident angle distribution. The specularity of the boundary is p = 0.9, meaning that 90% of the electrons are specularly reflected upon impact with the boundary and the remaining 10% are assigned a random angle. During the simulation, all points of impact with the boundary for all particles are saved and displayed in the histogram above. For further analysis, the number of impacts in the orange shaded regions, corresponding to the TEF peak areas, is extracted.

b) The counts in the orange shaded area in panel a as a function of TEF peak number are plotted in color for a wide range of simulated specularity coefficients p between 0 and 0.9. The black line is a fit of the form $Aq^x + c$ to the data, where A scales the overall amplitude, q is the extracted "experimental specularity coefficient" and c is a background offset.

c) The extracted experimental amplitude ratio q as a function of the specularity coefficient p for the 3-beam (orange) and 2-beam (blue) direction as well as a circular FS (purple). The black line indicates where q=p.

The main result of these simulations is that the assumption of q = p, identifying the true surface specularity p with the measured power law coefficient q is not strictly applicable, even for a circular Fermi surface. Hence the amplitude ratio $\frac{A_{n+1}}{A_n}$ is an indicator, but not a perfect measure of the specularity of the boundary. The physical reason for this is two-fold. In the case of fully specular reflection the peak width grows with the number of peaks and due to their convolution with a finite nozzle size the measured voltage decreases with increasing peak number. In the opposite limit, even in the case of completely diffusive boundary scattering (p=0), a large number of TEF peaks are expected to arise from focusing. Therefore, a simple analysis will extract a significant q value for p=0. Indeed, we find q≈0.33 for fully diffuse scattering (figure S7c). This value has a simple physical interpretation. Due the 3 main directions of propagation, approximately 1/3 of the electrons will be scattered into the direction that will be focused again. This statistical mechanism will lead to an apparent specularity of the boundary despite a completely diffusive scattering process. This is an alternative formulation of the super-geometric focusing properties of $PdCoO_2$.

Supplementary Note 8. Peak position analysis

In the following we analyze the ratio between the fields B_1 at which the first TEF maxima along the 2-beam and 3-beam direction occur, both for the experimentally measured and simulated spectra displayed in figure 3. For a mathematically hexagonal Fermi surface, no focusing is expected in the 2-beam-direction as discussed in the main text. Instead, here the ratio of the longest possible trajectories gives a measure of the range of electron transmission (Fig. S8). In the case of the ideal hexagon, the ratio of travel distances is geometrically given by the ratio of the inner to outer diameter of a hexagon, $\frac{\sqrt{3}}{2} \approx 0.87$. This simple argument is already quite close to the experimental results. When the rounded corners of the realistic Fermi surface of $PdCoO_2$ are taken into account, a macroscopic density of states will be focused at the same field, and a focusing peak occurs even in the 2-beam direction. The ratio of the furthest travelled distance in both directions increases to 0.91 for the realistic Fermi surface parameters obtained by ARPES and quantum oscillations. This happens as the distortions increase r_{3-beam} and decrease r_{2-beam}. In a circular Fermi surface, this ratio is naturally one and all TEF is independent of the crystal orientation. Note that a ratio closer to one, however, does not indicate a deformation to a more circular orbit, but merely reflects the geometric properties of the slightly star-shaped Fermi surface. This is exemplified in Fig. S8, as the realistic Fermi surface is distorted distinctly into a star-shape and not a more circular object. Yet the ratio increases. This back-of-the-envelope estimate already quite accurately reflects the ratio of the measured focusing fields.

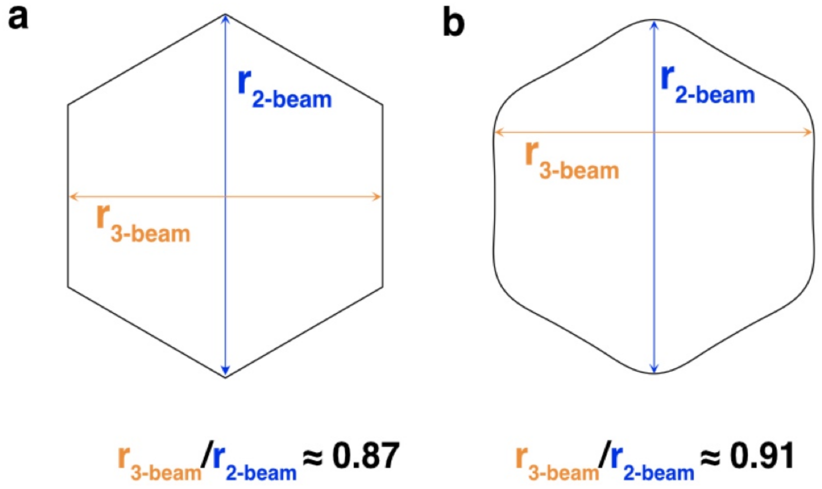

Supplementary Figure 8: Sketches of real space orbits indicating the maximal travel distance between nozzles, both for (a) the ideal hexagon and (b) the realistic Fermi surface.

To further improve the modelling, we take both the realistic Fermi surface and the finite nozzle width into account in the Monte-Carlo simulations. Here we obtain a ratio of 0.96 for the 2μm nozzle, in good quantitative agreement with the measured ratio of 0.99. The main reason for the increase of the ratio is that finite nozzles allow focusing trajectories over a small range of cyclotron radii, a geometric property that a full kinematic simulation naturally takes into account. The main factors attributing to the subtle deviation between experiment and tuning-parameter-free simulations (~3%) are measurement errors in the exact nozzle geometries in the SEM images;

nozzle-to-nozzle deviations of their width during fabrication; and the emission characteristic of the nozzles. The latter were assumed to be isotropic in k-space while in reality the ballistic connections to the nozzles themselves may lead to deviations in the emission spectrum.

	Simulation			Experiment		
Nozzle separation	2-beam direction	3-beam direction	ratio	2-beam direction	3-beam direction	ratio
2µm	6.19 T	5.96 T	0.96	6.04 T	6.00 T	0.99
4µm	3.23 T	2.98 T	0.92	3.18 T	3.07 T	0.965

Supplementary Table 2: Comparison of the ratio of the magnetic fields at which the first TEF maximum occurs for both the simulated and experimentally measured TEF spectra.

Supplementary Note 9. Extraction of the mean-free-path λ

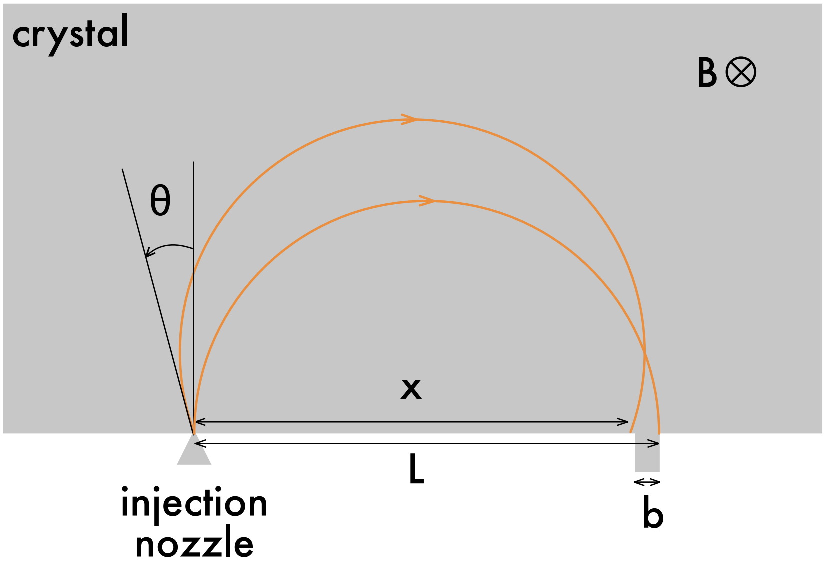

Supplementary Figure 9: Geometrical model of TEF on a circular FS.

The further two nozzles are spaced apart along the edge of the sample, the longer the path length s of an electron traveling through the bulk of the device, which increases the chances of being scattered away from its ballistic orbit. As pointed out by Tsoi *et al.*[34], the amplitude A_1 of the first TEF peak is proportional to $e^{-s/\lambda}$. The amplitude, however, also depends upon the ratio of b/L, where b is the width of the accepting nozzle and L is the distance between the nozzles. Assuming a point-like injection source and only the accepting nozzle having a width b, we find $x + b = L$, where L is the maximum distance an electron can travel at a fixed field ($L = 2r_c$). In a

system with a circular FS, the travel distance x of an electron injected under an angle **θ** can be found by trigonometry to be $x = 2r_c \cos\theta$, where $r_c = \frac{\hbar k_F}{eB}$ denotes the cyclotron radius and k_F is the Fermi momentum. With that and by Taylor expanding $\cos\theta \approx 1 - \frac{\theta^2}{2}$ for small θ, we find $\Delta\theta = 2\theta \approx 2\sqrt{\frac{2b}{L}}$. Accordingly, the amplitude of the first peak will decrease with increasing nozzle distance as $A_1 = 2\sqrt{\frac{2b}{L}} e^{-s/\lambda}$, where the path length is given by $s = \pi r_c$.

In the case of a hexagonal Fermi surface, the amplitude is similarly dependent on $e^{-s/\lambda}$ as well as the ratio b/L. For fitting the peak decay and extracting the mean-free-path **λ** in Fig. 3c we use the form $A_1(\mathrm{L}) = \mathrm{A}\ e^{-s/\lambda}\sqrt{\frac{b}{L} + t}$, with $b = 0.3\mu m$ and $s_{2beam} = \frac{3}{2}L$, $s_{3beam} = \sqrt{3}L$ are the path lengths for the 2-beam and 3-beam directions respectively. In addition to **λ**, the free variables are *A*, which sets the overall amplitude, and *t*, which takes the geometrical deviations from a non-circular FS into account. In the 3-beam direction the path-length is ill-defined due to the very nature of the super-geometric focusing effect. We choose the average between the longest and shortest path possible. The fit results are summarized in table 1. We note that this analysis is only valid for $\frac{b}{L} \ll 1$; once the nozzle width becomes comparable to the nozzle spacing the description breaks down. Further, particularly noticeable in the regime where $b \sim L$, but true in general, is that the maximum of the focusing peak does not occur at strictly $L = 2r_c$ but at lower magnetic fields when a nozzle of finite width can collect the maximum number of electrons.

	A [mΩ]	t [-]	λ [µm]
2-beam direction	7.7	-0.0037	14
3-beam direction	11.24	0.06	15.7

Supplementary Table 3: Free parameters for fitting the peak decay of A_1 as a function of nozzle distance (c.f. Fig. 3c) with the form $\mathrm{A_1(L) = A\ e^{-s/\lambda}\sqrt{\frac{b}{L} + t}}$. The small value of t in the 2-beam direction reflects the fact that the focusing in this orientation originates from the rounded corners of the hexagon which can be locally approximated by a circle. In the super-geometric focusing configuration the flat sides of the hexagon no longer resemble a circle leading *to a larger t value.*

Supplementary Note 10. Derivation of the TEF spectrum of a circular FS

In classical probability theory, let X and Θ be continuous variables, where $X = g(\Theta)$. The probability density function $f_\Theta(\theta)$ describes the probability of Θ falling within the infinitesimal interval [θ, θ+dθ]. This can be transformed according to $f_X(x) = f_\Theta(g^{-1}(x)) \cdot \left|\frac{d}{dx} g^{-1}(x)\right|$, which describes the probability of X falling into the interval [x, x+dx], in terms of the density if θ.

Let us consider the case of an (i) uncollimated and (ii) collimated beams of electrons injected into a TEF device. In all cases $x = g(\Theta) = 2r_c \cos\theta$ is the travelling distance of electrons when injected at x=0.

(i) For an uncollimated beam θ has a uniform density on $[-\frac{\pi}{2}, \frac{\pi}{2}]$

$$f_\Theta(\theta) = \begin{cases} \frac{1}{\pi} & for\ \theta\ \in \left[-\frac{\pi}{2}, \frac{\pi}{2}\right], \\ 0 & otherwise. \end{cases}$$

Requiring the probability density to be normalized, $\int_{-\infty}^{\infty} f_X(x)dx = 1$, we find: $f_X(x) = \frac{2}{\pi}\frac{1}{\sqrt{4{r_c}^2 - x^2}}$, corresponding to the curve shown in figure S4b.

(ii) Similarly, for a beam which is collimated in a cosine form, we find its density

$$f_\Theta(\theta) = \begin{cases} \frac{1}{2}\cos\theta & for\ \theta\ \in \left[-\frac{\pi}{2}, \frac{\pi}{2}\right], \\ 0 & otherwise. \end{cases}$$

The probability density function is then given by

$$f_X(x) = \frac{x}{2r_c}\frac{1}{\sqrt{4{r_c}^2 - x^2}} .$$

Supplementary Note 11. Numerical Methods

We start with a tight binding approximation of the FS[14] based on ARPES data[32],

$$\boldsymbol{k_F}(\theta) = k_0 + k_6\cos(6\theta) + k_{12}\cos(12\theta)$$

where $\mathrm{k}_0 = 0.95\dot{\mathrm{A}}^{-1}$, $\mathrm{k}_6 = 0.05\ \dot{\mathrm{A}}^{-1}$, and $k_0 = 0.006\ \dot{\mathrm{A}}^{-1}$. The equations of motion for an electron in an out-of-plane magnetic field $\boldsymbol{B} = B\hat{\boldsymbol{z}}$ are

$$\hbar v = \frac{\partial \varepsilon}{\partial k}, \qquad \hbar\dot{\boldsymbol{k}} = -e\boldsymbol{E} + eB\hat{z}\times\boldsymbol{v}$$

where $\hbar$ is the reduced Planck's constant, e is the charge of an electron, $\boldsymbol{v}$ is the Fermi velocity, and E is the electric field experienced by the electron. In the ballistic regime, there is negligible electric field in the bulk, therefore we assume that $E = 0$. As discussed in the main text, the real space trajectory is a 90° rotation of the FS scaled by a factor of $\hbar/eB$. Because we are not concerned with transit times of the electrons, we can ignore the Fermi velocity v.

When interacting with an edge of the device, the probability of injecting into a particular state n of the discretized Fermi surface is

$$p(n) = cos(\theta(n) - \phi)$$

where $\theta(n) = tan(v_y/v_x)$ is the direction of propagation of the state n and ϕ is the angle of the normal to the edge. The Fermi surface is numerically discretized into states separated by constant arclength to remove the probability distribution's dependence on Fermi velocity[1]. The nearly perfectly hexagonal Fermi surface of $PdCoO_2$ has approximately flat edges which cause a high density of states to be injected at fixed angles.

Charge carriers are injected into a simplified two-dimensional version of the $PdCoO_2$ TEF device, beginning at a random position along the injection ohmic contact in an allowed state of the discretized FS (Fig. S11). These carriers then follow their semi-classical path[1], ignoring bulk scattering, until interacting with either an edge or ohmic contact of the device. In the case of a non-ohmic edge, a carrier is scattered into a new randomly state chosen according to the probability distribution for that edge. To ensure detailed-balance, floating voltage leads absorb an incident carrier and subsequently, the carrier is reemitted at a random position along the lead in a randomly chosen allowed state for that edge. The voltage at a lead is given by

$$V \propto \frac{\Phi_{\mathrm{contact}}}{L_{\mathrm{contact}}}$$

where Φ_{contact} is the number flux of carriers through the contact and L_{contact} is the length of the device perimeter contacted by the voltage lead.

Electrons propagate within the device until they are absorbed by a grounded ohmic contact. The simulations of Fig. 3b of the main text are comprised of 1001 magnetic field points, each consisting of 30000 charge carriers, where the voltage difference between a TEF and a reference voltage lead all normalized by the voltage at the injecting contact V_{source} is plotted. We observe qualitatively similar magnetic field dependence between this simulated ratio and the measured

resistance of the real device for both tested orientations of device geometry relative to the crystal axis.

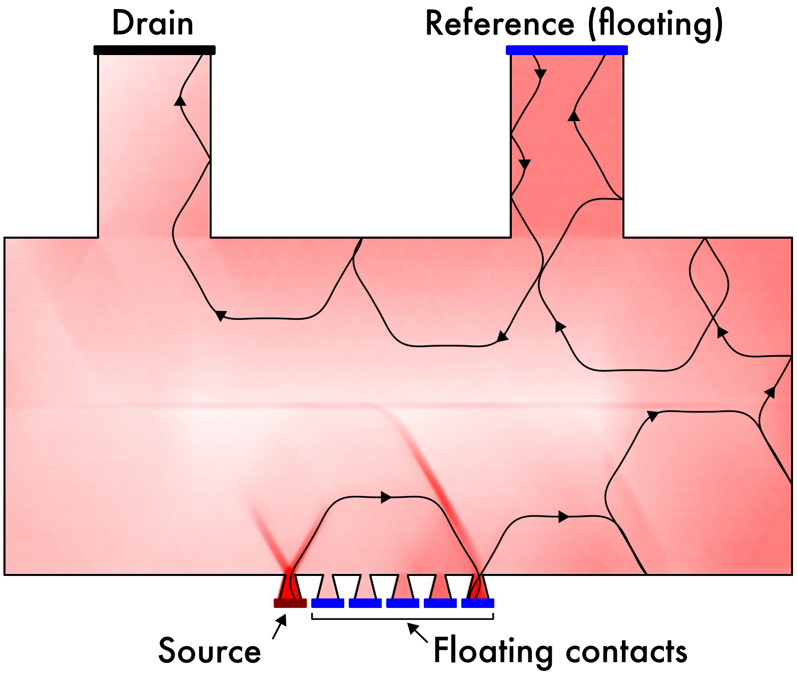

Supplementary Figure 11: Monte Carlo simulation of ballistic trajectories

Normalized heat map of the position of electrons in the simulated TEF device geometry. Real space is divided into a grid. A count for each plaquette is incremented when an electron's trajectory passes through that plaquette. This count is reflected in the tone of red, with darker red corresponding to a higher count (where the count has been cut off at a high number to provide contrast in the bulk of the device). An example of such a trajectory is shown in black. Electrons are injected at the source (maroon contact) and are propagated until hitting the drain (black contact). Electrons incident on floating contacts (blue) are absorbed and reinjected at a random point along the contact. The Fermi surface can be freely rotated relative to the device to simulate both the 2- and 3- beam orientations.
